# Comparison of vacuum-assisted sheaths and normal sheaths in minimally invasive percutaneous nephrolithotomy: a systematic review and meta-analysis

**DOI:** 10.1186/s12894-021-00925-1

**Published:** 2021-11-15

**Authors:** Ling Zhu, Zhenghao Wang, Ye Zhou, Liping Gou, Yan Huang, Xiaofeng Zheng

**Affiliations:** 1 Health Management Center, West China Hospital, Sichuan University, Chengdu, China; 2 Department of Endocrinology and Metabolism, Center for Diabetes and Metabolism Research, West China Hospital, Sichuan University, Chengdu, China

**Keywords:** Nephrolithiasis, Minimally invasive percutaneous nephrolithotomy, Vacuum-assisted sheath, Conventional sheath, Meta-analysis

## Abstract

**Background:**

A systematic review and meta-analysis was conducted to compare the safety and efficacy of vacuum-assisted sheaths and conventional sheaths in minimally invasive percutaneous nephrolithotomy (MPCNL) in the treatment of nephrolithiasis.

**Methods:**

PubMed, Web of Science, Embase, EBSCO, and Cochrane Library databases (updated March 2021) were used to search for studies assessing the effect of vacuum-assisted sheaths in patients who underwent MPCNL. The search strategy and study selection processes were implemented in accordance with the PRISMA statement.

**Result:**

Three randomized controlled trials and two case-controlled trials that satisfied the inclusion criteria were enrolled in this meta-analysis. Overall, the stone-free rate (SFR) in patients who underwent vacuum-assisted sheaths was significantly higher than that in patients who underwent conventional sheaths (RR 1.23, 95% CI 1.04, 1.46, *P* = 0.02), with significant heterogeneity among the studies (I^2^ = 72%, *P* = 0.03). In terms of the outcome of complications, vacuum-assisted sheath could bring a benefit to the postoperative infection rate (RR 0.48, 95% CI 0.33, 0.70, *P* < 0.00001) with insignificant heterogeneity among the studies (I^2^ = 0%, *P* = 0.68). There was no significant difference in the blood transfusion rate (RR 0.35, 95% CI 0.07, 1.73, *P* = 0.17), with significant heterogeneity (I^2^ = 66%, *P* = 0.35). Three studies contained operative time data, and the results indicated that the vacuum-assisted sheath led to a shorter operative time (MD = − 15.74; 95% CI − 1944, − 12.04, *P* < 0.00001) with insignificant heterogeneity (I^2^ = 0%, *P* = 0.91).

**Conclusion:**

The application of a vacuum-assisted sheath in MPCNL improves the safety and efficiency compared to the conventional sheath. A vacuum-assisted sheath significantly increases the SFR while reducing operative time and postoperative infection.

## Background

Urolithiasis is the third most common disease of the urinary tract, and its prevalence has increased over the past decades [1]. The prevalence rate of kidney stones worldwide is approximately 1.7% to 8.8%, and kidney stones cost approximately $2.1 billion in 2020 alone [2]. Patients with nephrolithiasis often suffer from short-term complications such as acute renal colic, nausea, vomiting, and hematuria and long-term complications such as chronic renal failure and hydronephrosis [3]. Therefore, treatment of calculi has always been the focus of surgeons. For renal stones > 2 cm in size, the American Urological Association recommends percutaneous nephrolithotomy (PCNL) as the primary treatment [4]. Previous studies have shown that standard PCNL is a highly effective approach [5]. However, it is often associated with major complications such as bleeding with the need for blood transfusion, postoperative fever, and pneumothorax [6].

In 2001, minimally invasive percutaneous nephrolithotomy (MPCNL), which involves the use of a small access sheath (i.e., 20F or less) was introduced in clinical practice [7, 8]. Despite the popularity of MPCL due to the lower risk of trauma and morbidities, it suffers from certain drawbacks. The efficiency of extraction of stone fragments and dust is lower than that of standard PCNL. Furthermore, the size of the sheath and the force of irrigation can lead to a higher incidence of infections due to the rise in renal intraluminal pressure and limitations in lithotripsy equipment [9]. With the development of new technologies, vacuum-assisted sheaths have emerged. Stone fragments and irrigation fluid can be sucked out continuously and contemporarily in the gap between the scope and sheath. Some studies on this issue have been conducted recently, but the outcomes of the effect and efficiency of MPCNL with a vacuum-assisted sheath are unsettled.

To date, there is still a lack of high-level evidence. The present write-up, therefore, aims to systematically review and perform a meta-analysis of the current studies to investigate the effectiveness and safety of vacuum-assisted sheaths for the treatment of nephrolithiasis.

## Materials and methods

The systematic review and meta-analysis were carried out following the guidelines of the Preferred Reporting Items for Systematic Reviews and Meta-analysis (PRISMA) statement and the Cochrane Handbook for Systematic Reviews of Interventions [10]. Ethical approval and patient consent were not required, as all the analyses were performed in previously published studies.

### Literature search and selection criteria

We systematically searched relevant published articles in several databases, including PubMed, Embase, Web of Science, EBSCO, and the Cochrane Library, from inception to March 2021 with the following keywords: “percutaneous nephrolithotomy,” “minimally,” “sheath,” “evacuation,” and “suction.” The reference lists in the retrieved studies and relevant reviews were manually searched, and the above process was repeated to ensure that all eligible studies were identified.

The inclusion criteria were as follows: (1) the study design was a randomized controlled trial, (2) the patient had a history of kidney stones and underwent minimally invasive PCNL, (3) the intervention approach was a suctioning sheath versus a normal sheath, and (4) the entire text was available. Studies satisfying these inclusion criteria in all languages were included.

### Data extraction and outcome measures

Baseline information extracted from the original studies included the first author, year of publication, number of patients, patient age and sex distributions, type of calcium stone, detailed methods for the two groups, and the evaluation of evidence level. Data were independently extracted by two investigators, and discrepancies were resolved by consensus.

The primary outcomes were stone-free rate (SFR) and perioperative complications (including postoperative infection rate, blood transfusion rate, and perforation rate). Secondary outcomes were operative time and hospitalization.

### Quality assessment of individual studies

All assessments were performed independently by two researchers, with differences resolved by discussion. The methodological quality of each RCT was assessed according to the Jadad Score, which comprises the following three evaluation elements: randomization (0–2 points), blinding (0–2 points), and dropouts and withdrawals (0–1 points) [11]. One point was awarded for each element that was conducted and appropriately described in the original article. The total score varies from 0 to 5 points. An article with a Jadad score of ≤ 2 is considered to be of low quality, while a Jadad score of ≥ 3 indicates a high-quality study [12].

### Statistical analysis

Risk ratios (RRs) with 95% confidence intervals (CIs) were calculated for dichotomous outcomes. Heterogeneity was evaluated using the I^2^ statistic, with I^2^ > 50% indicating significant heterogeneity [13]. Sensitivity analysis was performed to evaluate the influence of a single study on the overall estimate by omitting one study in turn or performing subgroup analysis. The random effects model was used for meta-analysis. Owing to the limited number of included studies (< 10), publication bias was not assessed. Statistical significance was accepted at *P* < 0.05. All statistical analyses were performed using Review Manager Software Version 5.3 (The Cochrane Collaboration, Software Update, Oxford, UK).

## Result

### Literature search, study characteristics, and quality assessment

**Fig. 1:**
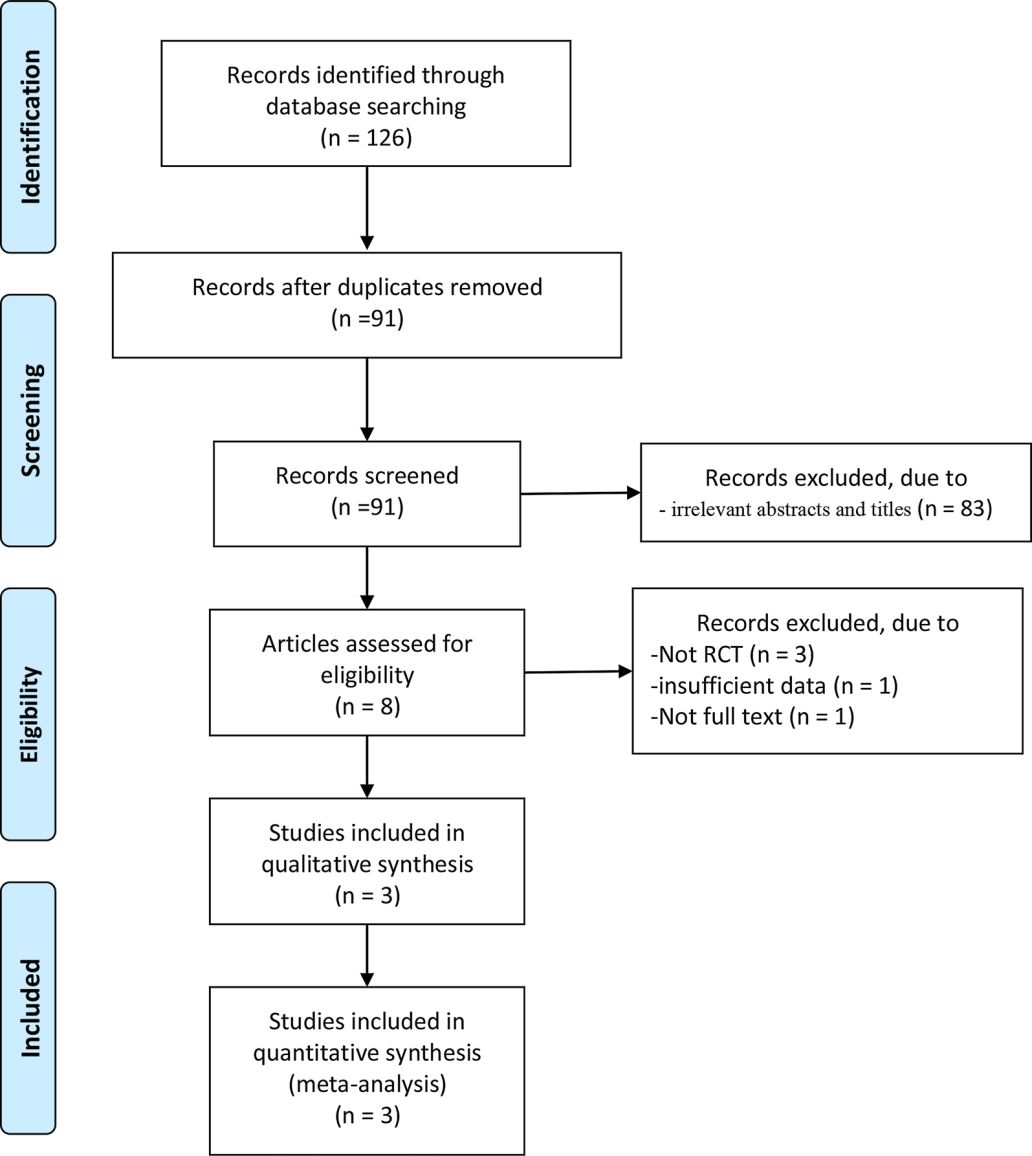
Flow diagram of the study search and selection process

A total of 126 articles were initially identified from the databases. After removing duplicates, 91 articles were retained. Then, 83 studies were excluded from the study due to unrelated abstracts and titles. One article with insufficient data, one article without full text, and three for not were also excluded from our analysis due to the study design. Finally, three randomized controlled trials (RCTs) with 857 patients who satisfied all inclusion criteria were subjected to the meta-analysis [14–16]. The article selection process was performed in accordance with the PRISMA statement (Fig. 1). The baseline characteristics of the three included studies are shown in Table 1. The studies included in this meta-analysis were published between 2016 and 2020. The JADAD score for three studies was 2, 3, and 4. One study was of low quality, as no blinding was used and the specific method of randomization was explained [14].

**Table 1:** Characteristics of included studies

No	Author	Year	Experimental group					Control group					Study design	Quality assessment
			Number (n)	Age (Mean ± SD)	Male (n)	Stone	Access sheath and lithotripsy energy	Number (n)	Age (Mean ± SD)	Male (n)	Stone size	Access sheath and lithotripsy energy		
1	Huang	2016	91	43.5 ± 2.9	53	Stone dimension 16.7 ± 5.8 mm	16F vacuum sheath/holmium laser	91	44.1 ± 3.2	51	15.1 ± 6.3 mm	16F peel-away sheath/holmium laser	RCT	3^a^
2	Du	2018	311	43.6 ± 17.4	187	Stone size 13.6 ± 5.2 cm^2^	16-18F vacuum sheath/ultrasound	304	41.2 ± 16.9	181	13.9 ± 4.7 cm^2^	16-18F peel-away sheath/ultrasound	RCT	2^a^
3	Xu	2020	30	52.1 ± 11.5	18	Stone diameter 4.2 ± 1.0 cm	20F vacuum sheath/pneumatic and holmium laser	30	51.8 ± 9.6	14	3.8 ± 1.4 cm	20F conventional sheath/pneumatic and holmium laser	RCT	4^a^

^a^Jadad score

### Primary outcomes

#### Stone-free rate

**Fig. 2:**
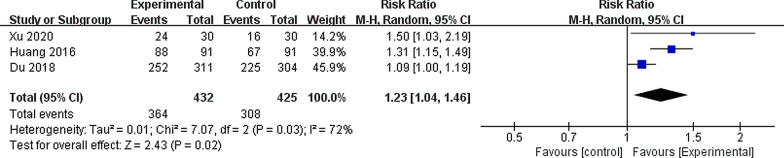
Forest plot for the meta-analysis of stone free rate

All studies included for the analysis reported the SFR, where stone-free was defined as stone fragments ≤ 4 mm (1 study using computed tomography (CT) scan and 2 studies using X-ray). Our results indicated that the SFR of the vacuum-assisted sheath group was significantly higher than that of the conventional sheath group (RR 1.23, 95% CI 1.04, 1.46, *P* = 0.02), with significant heterogeneity among the studies (I^2^ = 72%, *P* = 0.03) (Fig. 2).

#### Perioperative complications: postoperative infection rate, blood transfusion rate, and perforation rate

**Fig. 3:**
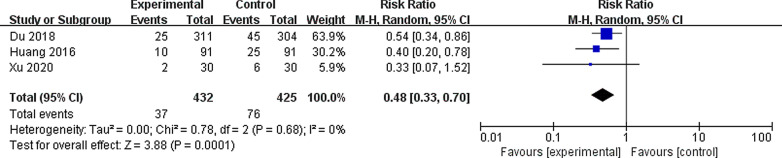
Forest plot for the meta-analysis of postoperative infection

**Fig. 4:**
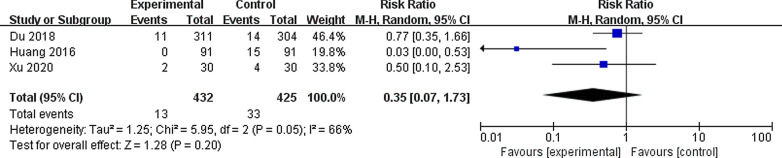
Forest plot for the meta-analysis of transfusion

All the studies reported infection-related complications and blood transfusion rates. Three studies reported postoperative fever [14–16]. These results indicated that vacuum-assisted sheaths provided a benefit to the postoperative infection rate (RR 0.48, 95% CI 0.33, 0.70, *P* < 0.00001), with insignificant heterogeneity among the studies (I^2^ = 0%, *P* = 0.68) (Fig. 3). There was no significant difference in the blood transfusion rate (RR 0.35, 95% CI 0.07, 1.73, *P* = 0.17), with significant heterogeneity (I^2^ = 66%, *P* = 0.35) (Fig. 4) reported in the three studies [14–16]. Only one study reported perforation, and the vacuum-assisted sheath had a higher incidence of perforation (7/91 vs. 1/91; *P* < 0.001).

### Secondary outcomes

#### Operative time

**Fig. 5:**
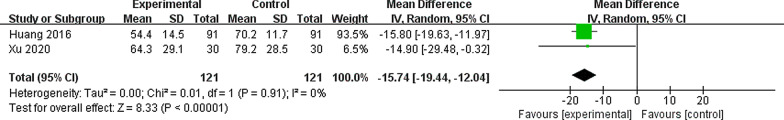
Forest plot for the meta-analysis of operative time

Three studies containing operative time data [15, 16] were analyzed, and the results indicated that the operative time of the vacuum-assisted sheath group was significantly reduced compared to that of the conventional sheath group (MD = − 15.74; 95% CI − 1944, − 12.04, *P* < 0.00001), with insignificant heterogeneity (I^2^ = 0%, *P* = 0.91) (Fig. 5).

#### Hospitalization

Only studies reported hospitalization data, and the results showed that vacuum-assisted sheaths might prolong the hospitalization time compared to normal sheaths (10.6 ± 3.7 vs. 6.4 ± 2.3; *P* < 0.001).

## Discussion

PCNL has been accepted as the gold standard for the treatment of large renal stones and is widely used in clinical practice [3, 17]. Although technological advances have ensured much progress in this field, many complications still exist [18]. To improve the safety and efficacy of this procedure, a small-size sheath was invented with the advent of mini-perc technology [19]. Due to the smaller size of the sheaths, MPCNL is associated with flaws such as longer operative time and infectious complications [20]. Recently, a sheath with irrigation and suctioning systems has been developed that can allow continuous infusion with saline intraoperatively [21]. Vacuum aspiration can be regulated manually or mechanically to keep the collecting system under negative pressure. Additionally, the nephroscope moves in and out conveniently through the movable sealing lid while preventing extremely high or low pressure [22]. To evaluate the effect and efficacy of the vacuum-assisted sheath, this meta-analysis was performed.

SFR is the main parameter for judging the efficacy of minimally invasive stone removal surgery [23]. Despite the difference in imaging modalities and follow-up time in the definition of SFR, our results show that vacuum-assisted sheaths have an improved stone clearance compared to normal sheaths. One possible explanation may be the low positive or low negative state of intrapelvic pressure controlled by the sheath while flushing and irrigation. In this situation, the kidney parenchyma shrank, tension in the renal pelvis decreased, and renal parenchymal compliance improved. Thus, the nephroscope can reach more renal calyces. Furthermore, when the calyceal neck is narrow or the angle is hard to reach, this sheath can perform lithotripsy and simultaneously suction out the fragments, making it a one-step procedure. Additionally, continuous negative pressure suction ensures a clear surgical field to avoid missing stone fragments, and therefore, a higher SFR is reached [14].

Xu et al. reported a higher incidence of postoperative fever [15]. Due to the small size of the sheath, high-pressure perfusion is very often performed. It is known that the limit of renal intrapelvic pressure is 30 mmHg [24]. Higher pressure can injure the integrity of the pelvic wall epithelium, leading to exposure of the lymphatic and venous systems [25]. In addition, tissue edema and congestion caused by urinary tract infection and stones are more likely to cause pelvic fluid absorption. When the bacteria along with the associated toxin reflux are absorbed, it may lead to infectious complications such as postoperative fever or sepsis [26]. By using the suction sheath, the renal pelvis is kept in a negative pressure state. Therefore, the infectious fluid flows smoothly, and the absorption of irrigation, bacteria, and toxin reflux is reduced. Du et al. found that MPCNL with a vacuum sheath has a low intrapelvic pressure compared to standard PCNL and MPCNL [14]. Xu et al. found that intrapelvic pressure ≥ 30 mmHg was achieved in the non-vacuum-sheath group [15].

Prolonged operative time is another independent risk factor for infectious complications [27]. The smaller size of access causing a limitation for more options for lithotripsy is a major inherent limitation of MPCNL. Another limitation is the small visual field in miniature endoscopes, which leads to a prolonged time to break the stones into smaller fragments [28]. Despite the different definitions of the operation time, present evidence indicates that vacuum-assisted sheaths can significantly shorten the operation time. The vacuum-assisted sheath could simultaneously suck out the small clots and fragments of stone in the gap. Furthermore, clearer vision was achieved during the procedure to shorten the surgery time [29]. Although a shorter operation time may decrease blood loss, this was not proven to be the case in our study, as it depends on many factors, such as puncture site, number, and size of the sheath [30].

Admittedly, there are a few limitations to this study. First, only three randomized controlled trials were included in our study, and the geographical region was single. Second, the impact of differences in puncture kidney calices and depth in sheath placement was not assessed in the included studies. Finally, there are some unpublished data and missing negative data in the original reports, and because of this publication bias, our conclusion may be skewed.

The application of a vacuum-assisted sheath in MPCNL improves the safety and efficiency compared to the conventional sheath. A vacuum-assisted sheath significantly increases the SFR while reducing operative time and postoperative infection. Due to the inherent limitations of the included studies, large-scale, multiregion, multicenter, prospective RCTs should be performed in the future to validate our results.

## Data Availability

The data and materials can be obtained by contacting the corresponding author.

## Abbreviations

MPCNL: Minimally invasive percutaneous nephrolithotomy
SFR: Stone-free rate
PCNL: Percutaneous nephrolithotomy
PRISMA: Preferred Reporting Items for Systematic Reviews and Meta-analysis
MINORS: The Methodological Index for Nonrandomized Studies
CIs: Confidence intervals
RCTs: Randomized controlled trials
CCTs: Case-controlled trials
CT: Computed tomography
